# Spontaneous rupture during observation for degenerated uterine leiomyoma revealing uterine leiomyosarcoma: a case report

**DOI:** 10.1093/bjrcr/uaaf023

**Published:** 2025-04-27

**Authors:** Masaki Tsuda, Hiromi Edo, Yuki Arai, Kohei Shikata, Ryo Tanaka, Tsubasa Ito, Morikazu Miyamoto, Masashi Takano, Chikako Sato, Sho Ogata, Hiroshi Shinmoto

**Affiliations:** Department of Radiology, National Defense Medical College, Tokorozawa, Saitama 359-8513, Japan; Department of Radiology, National Defense Medical College, Tokorozawa, Saitama 359-8513, Japan; Department of Radiology, National Defense Medical College, Tokorozawa, Saitama 359-8513, Japan; Department of Radiology, National Defense Medical College, Tokorozawa, Saitama 359-8513, Japan; Department of Radiology, National Defense Medical College, Tokorozawa, Saitama 359-8513, Japan; Department of Obstetrics and Gynecology, National Defense Medical College, Tokorozawa, Saitama 359-8513, Japan; Department of Obstetrics and Gynecology, National Defense Medical College, Tokorozawa, Saitama 359-8513, Japan; Department of Obstetrics and Gynecology, National Defense Medical College, Tokorozawa, Saitama 359-8513, Japan; Department of Laboratory Medicine, National Defense Medical College Hospital, Tokorozawa, Saitama 359-8513, Japan; Department of Laboratory Medicine, National Defense Medical College Hospital, Tokorozawa, Saitama 359-8513, Japan; Department of Radiology, National Defense Medical College, Tokorozawa, Saitama 359-8513, Japan

**Keywords:** uterine leiomyosarcoma, tumour rupture, diffusion-weighted imaging (DWI), apparent diffusion coefficient (ADC), intratumoural haemorrhage

## Abstract

This case report describes a 47-year-old premenopausal woman who presented with abdominal discomfort and had been previously monitored for a suspected uterine leiomyoma. MRI revealed a 15 cm mass within the uterine body and slight intratumoural haemorrhage. One month later, the patient presented with acute abdominal pain and was admitted to the hospital. Contrast-enhanced CT and MRI scans showed significant tumour enlargement to 20 cm, with disruption along the left margin, haemorrhagic ascites, and potential dissemination to the omentum. Total hysterectomy, bilateral salpingo-oophorectomy, and retroperitoneal lymph node dissection revealed uterine leiomyosarcoma with extensive necrosis and rupture. Pathological examination classified the tumour as stage IIB under the International Federation of Gynecology and Obstetrics system, with confirmed omental metastasis. Despite adjuvant chemotherapy, the patient experienced pelvic recurrence 10 months later and died 15 months postoperatively. This case emphasizes the importance of prompt gynaecological intervention for uterine masses exceeding 10 cm, as the risk of rupture increases, particularly when malignancy cannot be excluded based on imaging. Rupture in such cases is associated with a higher risk of recurrence and poor prognosis, making early surgical resection a reasonable consideration. Radiologists should actively communicate these risks to gynaecologists to facilitate timely surgical decision-making and improve patient outcomes.

## Clinical presentation

A 47-year-old premenopausal woman initially first visited her doctor 4 months prior with complaints of lower abdominal discomfort. Her medical history and medication records showed no notable findings. She was monitored for a suspected uterine leiomyoma. Two months later, she began experiencing atypical genital bleeding and was prescribed progestin medication.

## Investigations/imaging findings

**Figure 1.**
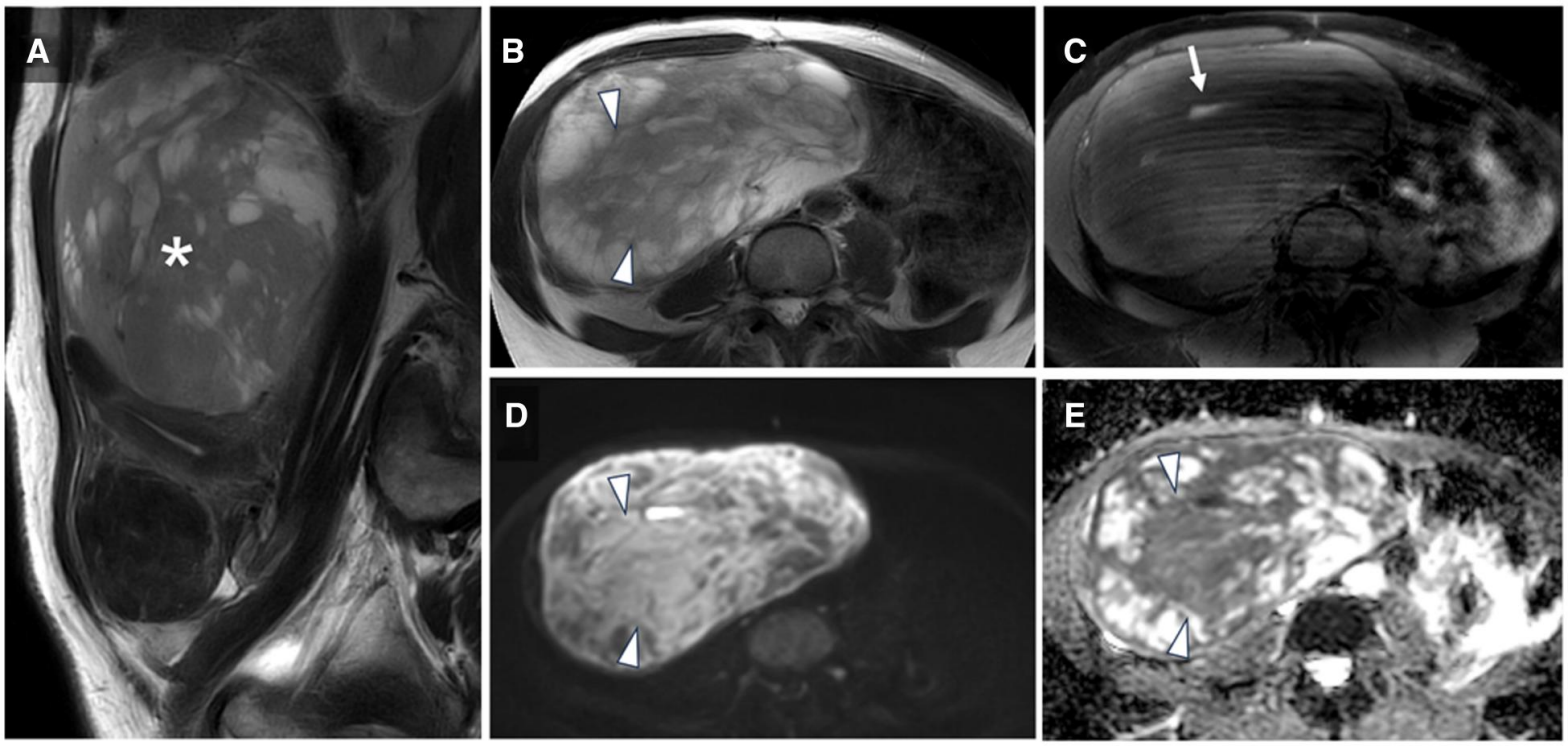
Initial MRI findings from the previous hospital. (A) Sagittal T2-weighted image (T2WI) showing a subserosal mass (*) with a maximum diameter of 15 cm located in the posterior wall of the uterus. The mass demonstrates mild hyperintensity and contains multiple cystic changes. (B) Axial T2WI showing a mildly hyperintense mass with cystic changes in the right lower abdomen. (C) Axial fat-suppressed T1-weighted image showing areas of hyperintensity within the mass, suggesting haemorrhage (white arrow). (D) Axial diffusion-weighted image (DWI) showing the mass with high signal intensity, consistent with its mild hyperintensity on T2WI, while the cystic areas appear hypointense. (E) Axial apparent diffusion coefficient (ADC) map showing the mass with low signal intensity, indicating restricted diffusion, while the cystic areas appear hyperintense, consistent with no restricted diffusion (white arrowhead).

MRI performed by her previous physician 1 month before her visit to our hospital showed a large 15 cm mass in the uterine body with heterogeneous hypersignal intensity on T2-weighted imaging (T2WI) (Figure 1A and B). Within the mass, a small hyperintense area was observed on fat-suppressed T1-weighted images, suggesting slight intratumoural haemorrhage (Figure 1C). The mass showed high signal intensity on diffusion-weighted imaging (DWI) and hypointensity on the apparent diffusion coefficient (ADC) maps in areas that showed slight hyperintensity on T2WI (Figure 1B, D, and E; white arrowheads). The mean ADC value in this region was 0.88 × 10^−3^ mm^2^/s.

The patient was admitted to our hospital with acute abdominal pain. The blood test results showed lactate dehydrogenase (LDH) 590 U/L, C-reactive protein 9.3 mg/dL, white blood cells 10.3 × 10^3^/dL, haemoglobin 8.7 g/dL, haematocrit 26.9%, and platelet 434 × 10^3^/dL. Elevated inflammatory markers and anaemia were observed, along with increased LDH levels.

**Figure 2.**
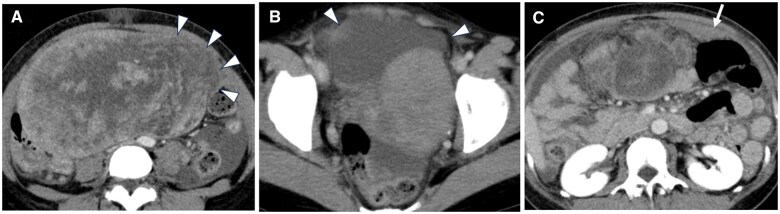
Contrast-enhanced CT at the time of admission due to abdominal pain (1 month after the initial MRI at the previous hospital). (A) The mass has enlarged to 20 cm. The mass is ruptured on the left side, with the contents of the mass protruding at the rupture site (white arrowheads). (B) Pelvic ascites are present, with some areas showing high density, indicative of haemorrhagic ascites. Peritoneal thickening is also noted. (C) At the upper abdominal level, disseminated lesions are observed in the omentum near the site of the mass rupture (white arrow).

Contrast-enhanced CT performed at our institution revealed a large uterine-origin tumour measuring 20 cm at its longest diameter (Figure 2), which had grown larger than the MRI conducted at a previous hospital a month earlier. Disruption was noted along the left margin of the tumour (Figure 2A; white arrowheads), and haemorrhagic ascites were present (Figure 2B; white arrowheads), suggesting abdominal pain due to tumour rupture. The absence of enlarged lymph nodes was indicative of no lymphatic metastasis. Additionally, a lesion suspected to have disseminated to the omentum was observed on the cranial aspect of the tumour (Figure 2C; white arrow).

**Figure 3.**
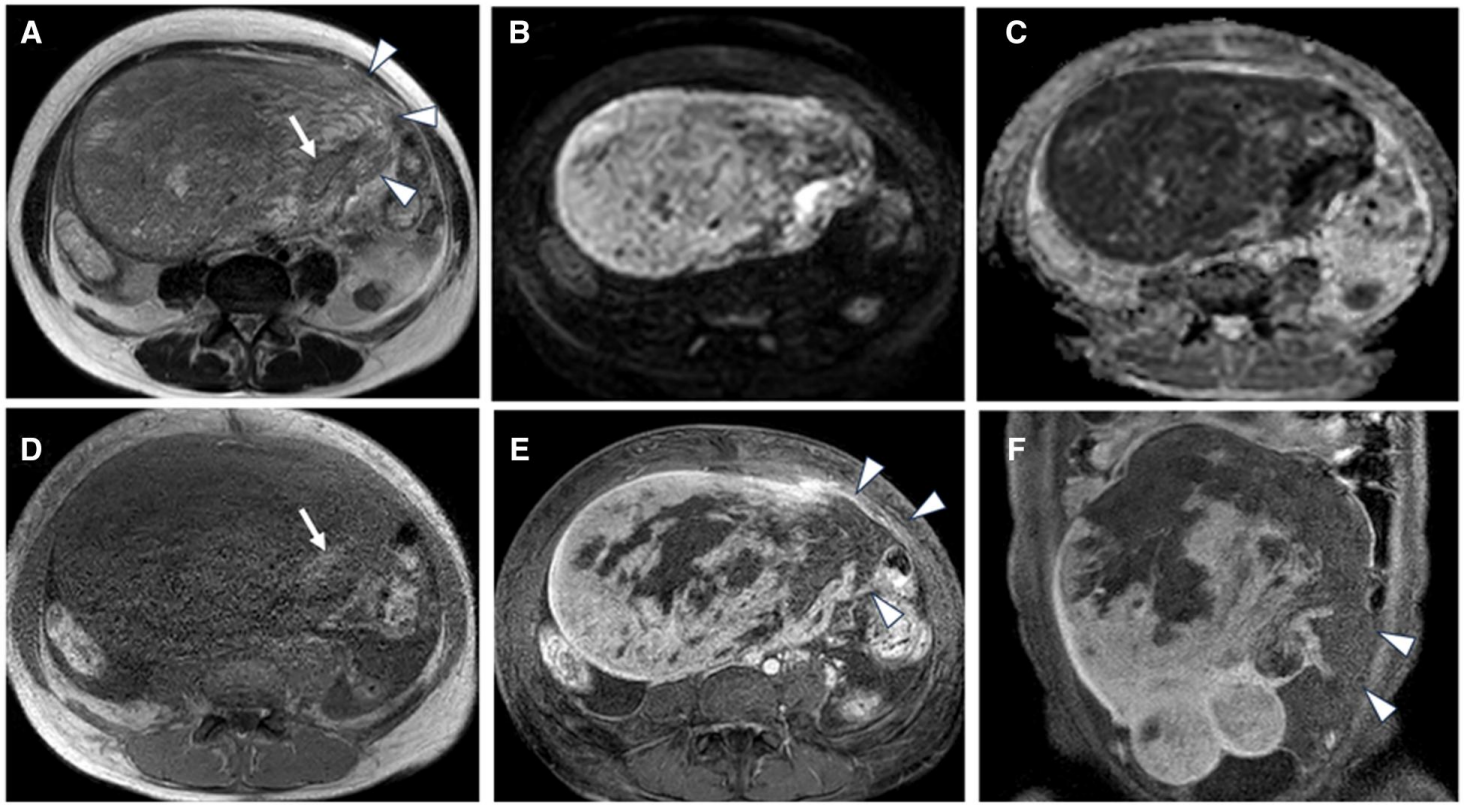
Contrast-enhanced MRI at the time of admission due to abdominal pain (1 month after the initial MRI at the previous hospital). (A) Axial T2-weighted image (T2WI) showing a mass measuring 20 cm in the right lower abdomen. The mass has increased in size with a higher proportion of solid components. The margin of the mass is disrupted on the left side, with protrusion of its contents at the disruption site (arrowhead). The mass demonstrates mildly high signal intensity with a low-signal-intensity rim (arrow). (B) Axial diffusion-weighted image (DWI) showing the mass with heterogeneous high signal intensity. (C) Axial apparent diffusion coefficient (ADC) map showing the mass with heterogeneous low signal intensity, indicating restricted diffusion. (D) Axial T1-weighted image showing high signal intensity within the mass (arrow), consistent with intratumoural haemorrhage. (E, F) Axial (E) and coronal (F) fat-suppressed contrast-enhanced T1-weighted images showing areas with poor enhancement within the mass, suggestive of necrosis, and disruption on the left side of the mass (arrowheads).

In the subsequent MRI, compared to the initial scan, the tumour demonstrated enlargement, an increase in the area of the contrast-enhancement defect suggestive of necrosis, and clearer evidence of intratumoural haemorrhage (Figure 3). Consistent with the CT findings, a rupture was observed on the left side of the tumour, accompanied by haemorrhagic ascites. However, the disseminated lesions noted on CT were indistinct on MRI, possibly due to field-of-view limitations.

## Differential diagnosis

The rupture of a uterine leiomyoma with degeneration or a malignant uterine tumour, such as a uterine sarcoma, was suspected.

## Treatment

The patient underwent a total hysterectomy, bilateral salpingo-oophorectomy, and retroperitoneal lymph node dissection. Intraoperative findings revealed a large tumour in the fundus of the uterus with extensive necrosis and rupture. Adhesions were observed in the omentum, and metastasis was suspected. The pathological diagnosis during surgery was uterine leiomyosarcoma, and the ascites cytology was negative.

**Figure 4.**
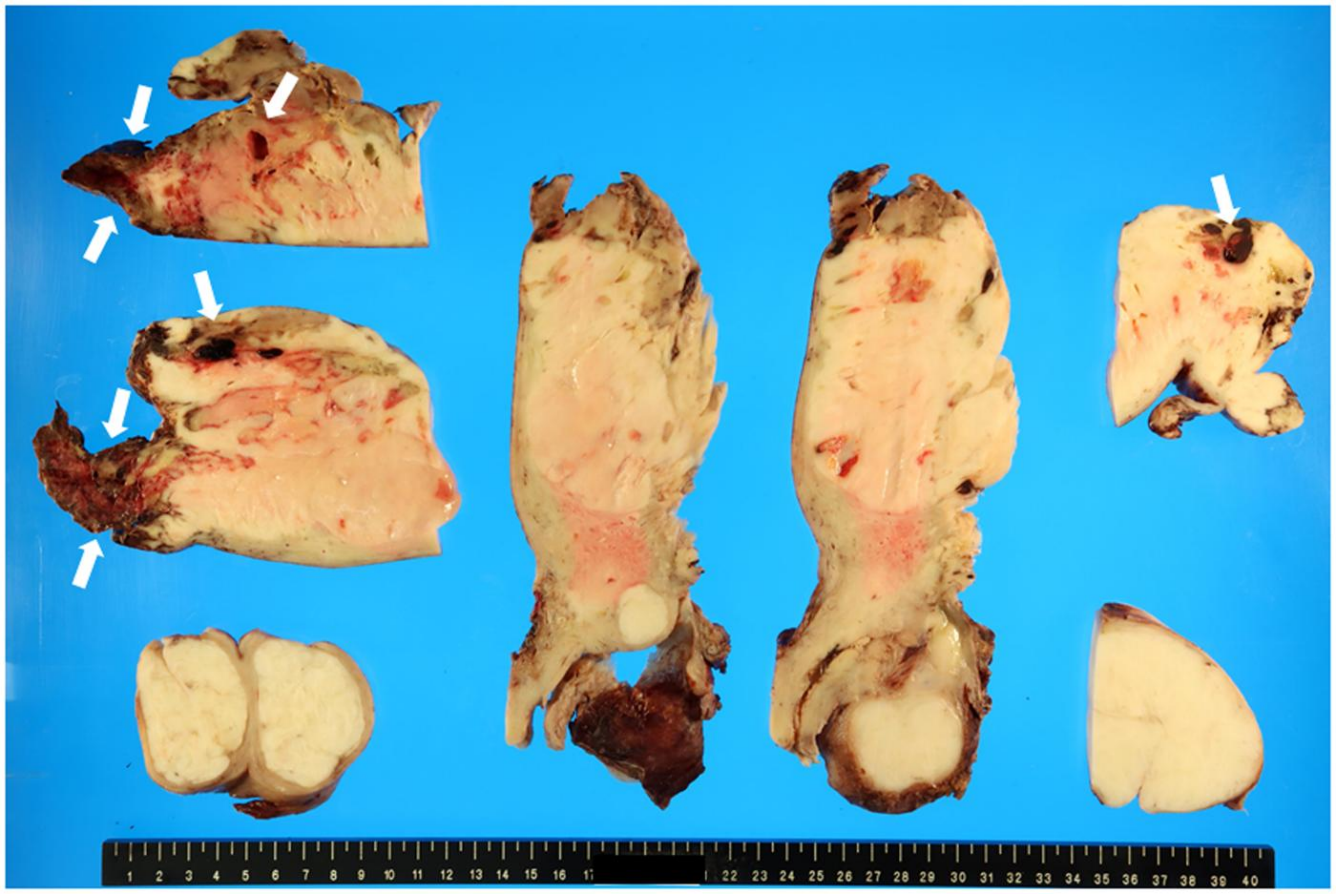
Macroscopic image of a removed uterine mass. A greyish-white dense mass is observed at the base of the uterus. The dark brown area inside the mass corresponds to a haemorrhage (arrows).

Macroscopic examination of the excised specimen revealed a uterine mass measuring 235 × 170 × 75 mm within the uterine fundus (Figure 4). The tumour appeared partially exposed on the serosal surface, with signs of rupture. The histopathological examination showed a proliferation of spindle-shaped tumour cells in the tumour that also resemble smooth muscle cells, corresponding to uterine leiomyosarcoma. Disseminated metastases were also observed in the omentum. This was equivalent to stage IIB, according to the International Federation of Gynecology and Obstetrics classification. No metastases were observed in the bilateral adnexa or lymph nodes.

Adjuvant chemotherapy with Adriamycin was administered to the patient.

## Outcome and follow-up

**Figure 5.**
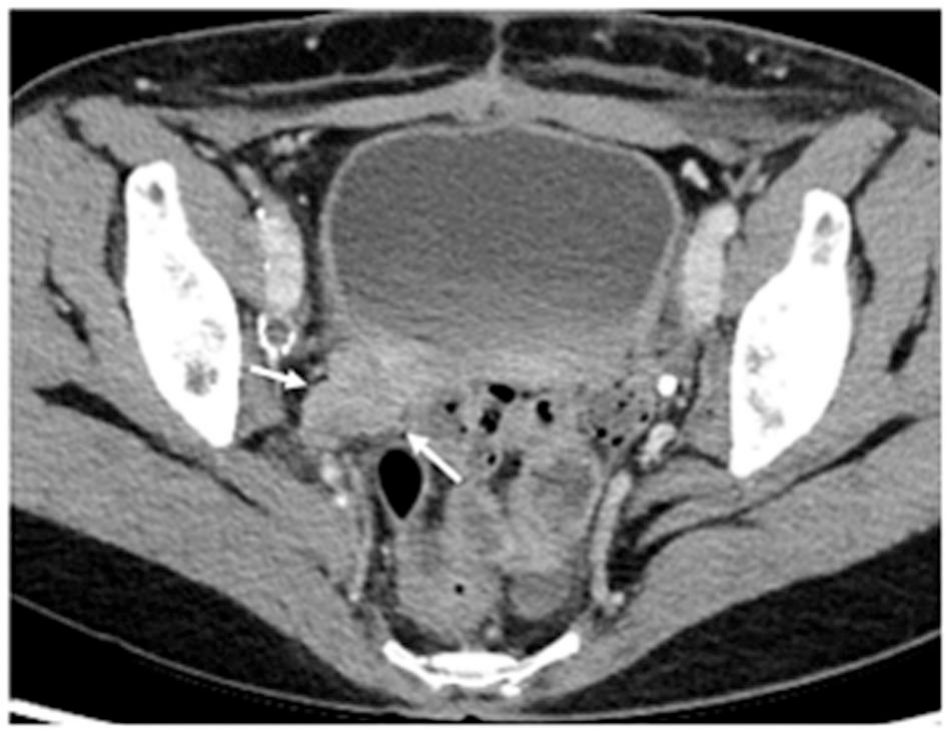
Contrast-enhanced CT at 10 months postoperatively. A 1.5 cm mass is observed on the right side of the pelvic cavity, with suspicion of disseminated recurrence (arrow).

A contrast-enhanced CT scan performed 10 months after surgery revealed disseminated recurrence in the pelvis (Figure 5), necessitating open tumour resection. The patient died 15 months after the initial surgery.

## Discussion

During the follow-up of a 15 cm uterine mass initially suspected as a degenerative uterine leiomyoma, CT and MRI were performed after abdominal pain due to rupture. Imaging revealed the rupture of an enlarged uterine mass, disseminated lesions, and haematoperitoneum.

Leiomyosarcomas are the most common subtype of uterine sarcomas, yet they account for only 1%-2% of malignant uterine tumours.^1^ Furthermore, ruptured uterine leiomyosarcoma is extremely rare, with only 9 cases reported in the past (Table 1).^2–10^ Ruptured uterine leiomyosarcomas have been reported in the size range of 5.8-11 cm, with all but one case being 8-11 cm in size, and the risk of rupture is considered to increase when the size exceeds 8 cm. The rupture mechanism is hypothesized to involve tumour growth that outstrips the blood supply, resulting in necrosis of surface blood vessels and subsequent rupture.^7^ Regarding the relationship between size and mass rupture in uterine leiomyomas, studies have reported that the risk of rupture increases when the size of the mass exceeds 10 cm, as the mass is accompanied by vascular stretching and tension.^11^ Additionally, once a mass ruptures, the risk of dissemination and recurrence increases. Of the 9 cases of ruptured uterine leiomyosarcoma, recurrence or metastasis developed within 1 year in 2 of the 4 cases with known postoperative outcomes, suggesting a poor prognosis (Table 1). Similarly, our patient experienced a poor prognosis, with pelvic recurrence occurring 10 months after the initial surgery and dying 15 months later.

**Table 1.** Published cases of ruptured uterine leiomyosarcoma.

Case	Author	Age (years)	Symptoms	Size (mm)	Surgical procedure	Metastasis/dissemination	Postoperative therapy	Recurrence and prognosis
1	Lazaro et al. 1980^2^	33	Abdominal pain	80	TAH + BSO	Douglas pouch dissemination	Chemoradiotherapy	No recurrence for more than 9 months
2	Barua and Olesnicky 1988^3^	50	Lower abdominal pain	80	TAH	Lung metastasis	No treatment	N/A
3	Farhi et al. 1993^4^	44	Syncope	110	TAH + BSO	None	Chemotherapy	N/A
4	Hicks et al. 2010^5^	58	Abdominal pain	110	TAH + BSO	None	N/A	N/A
5	Takeuchi et al. 2014^6^	59	Abdominal distension and pain	110	TAH + BSO	None	No treatment	No recurrence for more than 16 months
6	Boussouni et al. 2016^7^	43	Irregular uterine bleeding and pain	58	TAH + BSO	None	Chemoradiotherapy	N/A
7	Tanaka et al. 2022^8^	56	Abdominal pain	110	TAH + BSO + partial omentectomy and ileectomy	None	Chemotherapy	Lung metastases after 1 year
8	Kim et al. 2023^9^	58	Abdominal pain	90	TAH + BSO	Douglas pouch dissemination, pelvic lymph node metastases	Chemotherapy	Recurrence after 1 month and died after 7 months
9	Bicanin-Ilic et al. 2024^10^	49	Prolonged metrorrhagia and anaemia	110	TAH + BSO + omentectomy	Lung, liver, colon, and peritoneal metastases	Chemotherapy	N/A
10	This case	47	Abdominal pain	200	TAH + BSO	Omental metastasis	Chemotherapy	Recurrence after 10 month and died after 15 months

Abbreviations: BSO = bilateral salpingo-oophorectomy; N/A = not available; TAH = total abdominal hysterectomy.

Uterine leiomyosarcoma may present with clinical and imaging findings similar to those of degenerating uterine leiomyoma, a tumour derived from smooth muscle tissue. This similarity can make differentiation difficult. Uterine leiomyosarcomas are generally larger and grow more rapidly than uterine leiomyoma. Intratumoural haemorrhage and necrosis were characteristic findings. In this case, MRI after tumour rupture (Figure 3) showed an enlarged tumour and internal necrosis with a haemorrhage, and CT (Figure 2) showed lesions suggestive of dissemination. These findings led to the suspicion of uterine malignancy. However, at the time of the pre-rupture, MRI (Figure 1) showed minimal bleeding in the tumour, making it difficult to suspect uterine leiomyosarcoma at this stage.

In MRI, ADC values and the combination of T2WI with ADC/DWI have been reported to be useful for differentiating uterine leiomyomas from uterine leiomyosarcomas. Sato et al^12^ reported that the mean ADC value of uterine leiomyosarcoma lesions was significantly lower than that of uterine leiomyoma lesions, with 100% sensitivity and 94.0% specificity when the tumour showed moderate or high signal intensity on DWI and an ADC value of ≤1.1 × 10^−3^ mm^2^/s as the cut-off value. Namimoto et al^13^ reported that when the tumour shows higher signal intensity than the myometrium on T2WI and an ADC value of <1.05 × 10^−3^ mm^2^/s, it achieves 100% sensitivity and specificity for diagnosing uterine sarcomas. In DWI, lesions with high cell density are depicted as high signal intensity, and quantitative evaluation of ADC values is possible. ADC values typically reflect restricted diffusion and are reduced in cases of high cellular density. However, low signal intensity on T2WI can also influence ADC values, leading to falsely low measurements—a phenomenon known as the T2 blackout effect. Therefore, when ADC values are low, it is critical to distinguish whether this reflects true restricted diffusion due to high cellular density or is an artefact caused by low T2WI signal intensity. To make this distinction, careful evaluation of the signal intensity on T2WI in conjunction with ADC mapping is essential. Both may show similar low ADC values, but evaluating the tumour-to-myometrium signal ratio on T2WI helps resolve this overlap and improves diagnostic accuracy. In this case, the signal intensity of the tumour on T2WI before tumour rupture was higher than that of the myometrium. Additionally, the tumour showed a high signal intensity on DWI with an ADC value of 0.88 × 10^−3^ mm^2^/s (Figure 1). These findings were consistent with those reported by Sato et al and Namimoto et al. Notably, ADC values can vary depending on technical factors, including coil systems, vendors, imagers, and MRI parameter settings.^14^ Therefore, while ADC values are useful as a reference, they should not be used as the sole determinant; instead, careful interpretation in conjunction with other imaging features is necessary for accurate diagnosis.

MRI offers useful information for differentiating uterine leiomyosarcoma and uterine leiomyoma; however, complete differentiation by imaging alone remains challenging. Consequently, if a uterine mass larger than 10 cm with a high rupture risk does not present as a typical uterine leiomyoma and leiomyosarcoma cannot be excluded by MRI, radiologists should alert the gynaecologist to the malignancy potential and recommend early resection to prevent rupture.

## Conclusion

This case report highlights the importance of careful differentiation between benign and malignant uterine tumours, especially when the tumour exceeds 10 cm in size, as the risk of rupture and poor prognosis increases significantly. MRI findings, including ADC values, T2WI, and DWI, play a crucial role in assessing malignancy, but limitations in imaging alone necessitate strong clinical suspicion and timely intervention. Radiologists and gynaecologists must collaborate to ensure early resection when malignancy cannot be excluded, aiming to prevent rupture and improve patient outcomes.

## Learning points

- Large uterine masses (>10 cm) present a significant risk of rupture.
- MRI, including diffusion-weighted imaging/apparent diffusion coefficient value and T2-weighted image analysis, provides critical but not definitive differentiation between benign and malignant uterine tumours.
- Intratumoural haemorrhage and necrosis on imaging may raise suspicion of uterine leiomyosarcoma, even in cases resembling degenerative leiomyomas.
- Early resection is essential for preventing rupture and improving prognosis in cases of suspected malignancy.
